# Notes and News

**Published:** 1899-09-23

**Authors:** 


### NOTES AND NEWS.

(Collected and Communicated.)

The annual congress of the Royal Institute of Public Health commenced at Blackpool on Thursday, 21st, and will be continued until the 28th inst.

The Norwegian vessel "Taurus" put into Falmouth on Saturday on her way from Carabella, Florida, to Hamburg, having lost four men from sickness during the voyage, and several of the crew being ill. They were found to be suffering from beri-beri.

The Hospital Reform Association will hold a conference in St. Martin's Town Hall on October 10th and 11th. The subjects down for discussion are: "The Inquiry System," at four o'clock on the 10th; "Payments by Patients," on the same evening at eight p.m.; and "Provident Dispensaries," at four p.m. on October 11th.

The condemnation of fruit to be used in Lipton's jam manufactory, whilst meeting with general approval as a proof of the vigilance which is exercised in the inspection of food stuffs, will naturally raise some alarm also. Apparently jam made from such fruit as was examined was considered unwholesome, or the fruit would not have been condemned. If this is the case the public will conclude there is a great deal of unwholesome jam on the market, for the manufacturers who use unsound fruit to make jam must be very numerous seeing how very short a time fruit could have remained in good condition in such weather as we have experienced this summer.

The account we published last year of the little hoppers' hospital excited so much interest that our readers may be glad to know that a similar arrangement has been made this year. A cottage has been taken at Five Oak Green, near Tonbridge, and has already proved most useful. The children of hoppers are in especially bad circumstances if taken ill away from such comforts as they might secure even in their poor homes. So much has this been found to be the case, that Canon Carter, of Canterbury, has opened another hospital at Mereworth. We learn that the matron will gladly receive assistance for carrying on the work of these hospitals, and donations should be addressed to Sister Dowie, Little Hoppers' Hospital, Five Oak Green, Paddock Wood, Kent.

A point that is often misunderstood has been decided by Mr. McGee, revising barrister at Birmingham. This point is whether having received relief from the Guardians in the form of medical assistance, by residence in a workhouse infirmary as a patient, should disqualify a man from voting. Mr. McGee, in respect to a large number of cases recently at Birmingham, decided that it did not.

The Lunacy Commissioners summoned a workhouse attendant for unlawfully keeping a lunatic for payment. The defence was that Townsend, the attendant in question, was not aware that the man was a certified lunatic, and as Townsend had taken good care of him the magistrate dismissed the case. Townsend had definite charge of the lunatic in question, and the moment he showed violence he was removed to the workhouse.

In consequence of the continued spread of bubonic plague in India the India Office has appointed 20 medical men and 30 nurses for plague duty, the majority of whom have already left England on their way to Bombay. Many of the medical men who have gone out have already had experience of plague duty, and, taking them all round, they are an exceedingly well-qualified set of men. The India Office is to be congratulated on having been able to obtain so smart a set of medical volunteers at so short a notice.

In response to the request of the Liverpool School of Tropical Diseases that the American Government should attach an official delegate to the malarial commission of the school now working in West Africa, an answer has been received stating that it is regretted that the invitation was not received in season to have detailed an officer to accompany the expedition. The fact that the invitation was not received in time seems, however, to have been due to the expedition having started at extremely short notice, in order that Major Ross might have the full benefit of the rainy season in Sierra Leone.

Speaking of the difficulties which stand in the way of obtaining immediate isolation in cases of small-pox in some parts of the country, Dr. Edward Seaton, medical officer of health for the county of Surrey, says that the following is a case coming within his official knowledge and experience. A case of small-pox is imported into a common lodging-house, and is removed under compulsion to the workhouse. But its isolation at the workhouse is against official rules. It is therefore removed to an isolation hospital, where cases of scarlet fever, diphtheria, and typhoid are under treatment. Again this is against official rules. Yet when the sanitary authority in question endeavours to obtain a site for a small-pox hospital in one of the most lonely spots within thirty miles of London, its application is opposed by a resident within a mile of the place, and the authority has in the end to spend some thousands of pounds in defending this action. Fortunately at present there is not much small-pox about, but the management of this disease is a very serious difficulty to any sanitary authority which happens to have to undertake it, in consequence of the practical impossibility of obtaining a site for a small-pox hospital or of using it when obtained without running a risk of actions for nuisance.

